# Transcriptome and metabolite analyses provide insights into zigzag-shaped stem formation in tea plants (*Camellia sinensis*)

**DOI:** 10.1186/s12870-020-2311-z

**Published:** 2020-03-04

**Authors:** Hongli Cao, Feiquan Wang, Hongzheng Lin, Yijun Ye, Yucheng Zheng, Jiamin Li, Zhilong Hao, Naixing Ye, Chuan Yue

**Affiliations:** 1 College of Horticulture, Fujian Agriculture and Forestry University/Key Laboratory of Tea Science in Universities of Fujian Province, Fuzhou, 350002 China; 2 College of Tea and Food Science, Wuyi University, Wuyishan, 354300 China

**Keywords:** Auxin transport, Gravitropism response, Stem development, Tea plant, Zigzag-shaped stem

## Abstract

**Background:**

Shoot orientation is important for plant architecture formation, and zigzag-shaped shoots are a special trait found in many plants. Zigzag-shaped shoots have been selected and thoroughly studied in *Arabidopsis*; however, the regulatory mechanism underlying zigzag-shaped shoot development in other plants, especially woody plants, is largely unknown.

**Results:**

In this study, tea plants with zigzag-shaped shoots, namely, Qiqu (QQ) and Lianyuanqiqu (LYQQ), were investigated and compared with the erect-shoot tea plant Meizhan (MZ) in an attempt to reveal the regulation of zigzag-shaped shoot formation. Tissue section observation showed that the cell arrangement and shape of zigzag-shaped stems were aberrant compared with those of normal shoots. Moreover, a total of 2175 differentially expressed genes (DEGs) were identified from the zigzag-shaped shoots of the tea plants QQ and LYQQ compared to the shoots of MZ using transcriptome sequencing, and the DEGs involved in the “Plant-pathogen interaction”, “Phenylpropanoid biosynthesis”, “Flavonoid biosynthesis” and “Linoleic acid metabolism” pathways were significantly enriched. Additionally, the DEGs associated with cell expansion, vesicular trafficking, phytohormones, and transcription factors were identified and analysed. Metabolomic analysis showed that 13 metabolites overlapped and were significantly changed in the shoots of QQ and LYQQ compared to MZ.

**Conclusions:**

Our results suggest that zigzag-shaped shoot formation might be associated with the gravitropism response and polar auxin transport in tea plants. This study provides a valuable foundation for further understanding the regulation of plant architecture formation and for the cultivation and application of horticultural plants in the future.

## Background

In higher plants, the morphology of the plant mainly depends on the development and orientation of the shoots; therefore, plant shoots, which are usually negatively geotropic, play a crucial role in morphological architecture formation in many plants, such as landscape plants, fruit trees and other plants of economic interest. The growth and development of shoots are regulated by diverse factors, including light, temperature and phytohormones [1, 2], but gravitropism is important for the upward growth of shoots and is a determinant of the form and posture of plants [3–5]. However, the molecular mechanism by which shoot architecture formation in woody plants is mediated by gravitropism remains largely unknown.

In plants, gravity is sensed by specialized cells called statocytes, and then, a signal is transported to the elongation zone, leading to differential cell growth in the elongation zone to reorient organ growth in response to the gravity signal [6]. It is thought that sedimentable amyloplasts play a critical role in gravity perception and that endodermal cells containing sedimented amyloplasts function as statocytes in plant shoots [3, 7]. In *Arabidopsis*, shoot responsiveness to gravitropism is regulated by a group of SHOOT GRAVITROPISM *(SGR*) genes, of which at least nine *SGR* genes have been isolated and functionally studied [3]. It has been established that mutant *sgr* genes could reduce the plant gravitropism response by regulating endodermal cell development, starch accumulation and amyloplast movement [7–11]. Kato, et al. [8] and Morita, et al. [10] found that both *SGR2* and zigzag (*ZIG*)*/SGR4* were involved in the formation and function of the vacuole, suggesting that vacuolar integrity participates in shoot gravitropism. Interestingly, ZIG/SGR4 encodes Qb-SNARE VTI11, which is involved in membrane trafficking between the trans-Golgi network and the vacuole, and the stems of *zig*/*sgr4* mutants elongate in a zigzag fashion [8, 10, 11].

Plant shoot architecture formation is regulated by plant hormones, especially auxin gradients, which are integrated into almost all aspects of plant growth and development [12, 13]. Shoot curvature is controlled by auxin, and increased auxin levels promote cell elongation at the bottoms of reoriented shoots, resulting in upward bending. Therefore, the genes involved in auxin transport and response, especially the polarly localized PIN-FORMED (PIN) auxin efflux carriers, play crucial roles in plant shoot formation [13, 14]. PIN-mediated polar auxin transport has been well recognized as being involved in the plant gravity response [3, 7, 15]. For example, after gravistimulation, PIN3 and PIN7 are relocalized towards the gravity vector and trigger changes in auxin flux [16]. Recently, several individual genes, such as *LAZY1*, *LPA1*, and *WEEP,* have been characterized and recognized as new factors involved in plant architecture regulation; these genes also depend on auxin and gravity signalling [17–23], indicating that gravity response and polar auxin transport play primary roles in plant architecture development. However, previous reports have mainly focused on model plants, and the mechanism of architecture development in woody plants remains largely unknown.

The tea plant (*Camellia sinensis* (L.) O. Kuntze), which is native to southwestern China, is a perennial evergreen woody plant in the family *Theaceae* [24, 25]. Its tender leaves and buds are processed as a beverage for drinking because tea leaves contain secondary metabolites that are healthy for humans, and tea plants are currently cultivated in over 60 countries around the world [26, 27]. Numerous studies have examined secondary metabolism and stress-response mechanisms in tea plants; conversely, tea plant growth and development processes, such as architecture formation, which is one of the key regulators of tea yield [28], tea processing and even ornamental value, remain to be revealed. Two tea plant cultivars, namely, *C. sinensis* var. Qiqu (QQ) and *C. sinensis* var. Lianyuanqiqu (LYQQ), both with zigzag-shaped shoots, were selected and planted more than 80 years ago and described in a previous study [28], but the molecular mechanism of zigzag-shaped shoot formation remains unknown. In this study, the stems of tea plants with zigzag-shaped (QQ and LYQQ) and erect (*C. sinensis* var. Meizhan, MZ) shoots were collected and investigated for structural differences, transcription and metabolic regulation using section observation, transcriptome sequencing and metabolite detection, respectively, for the first time. In total, 46.06 million reads were generated from nine samples, and the differentially expressed genes (DEGs) were identified and compared. A total of 2175 DEGs, including 998 upregulated and 1177 downregulated genes, were identified from the zigzag-shaped shoots of QQ and LYQQ compared to the erect shoots of MZ. The candidate DEGs, including genes associated with vesicular trafficking, cell expansion, transcription factors, and phytohormones and diverse genes involved in the regulation of plant growth and development, were seemingly related to zigzag-shaped shoot formation in tea plant. The results will contribute greatly to an improved understanding of the molecular regulatory mechanism of zigzag-shaped shoot formation in woody plants.

## Results

### Phenotypic characterization and stem ultrastructure analysis of tea plants with erect and zigzag-shaped shoots

**Fig. 1:**
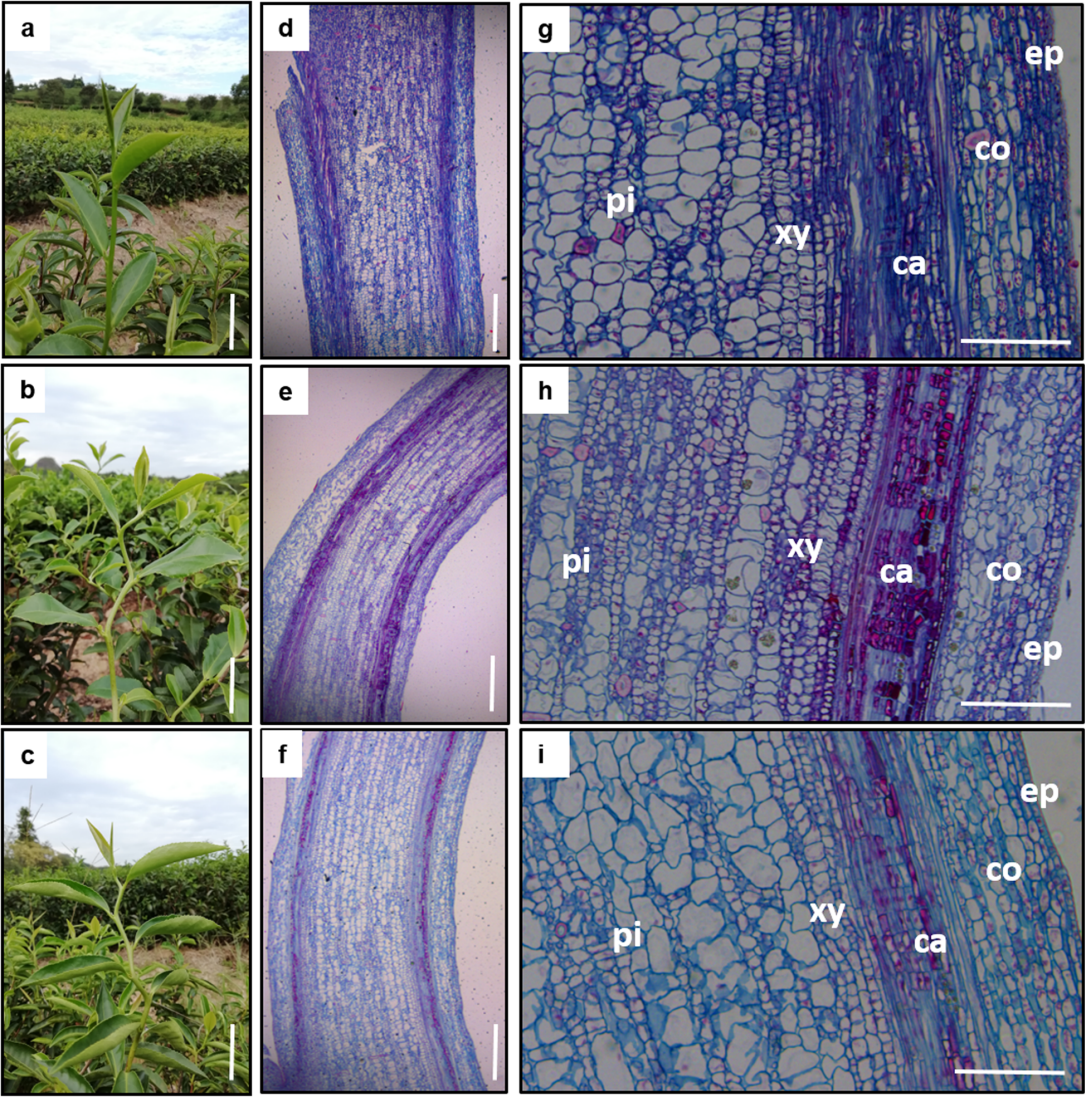
Morphology and histological analysis of the shoots of MZ, QQ, and LYQQ. **a** to **c**, Tender stem morphology of MZ (**a**), QQ (**b**), and LYQQ (**c**). Bars = 1 cm. **d** to **i**, Longitudinal sections of tender stems of MZ (**d** and **g**), QQ (**e** and **h**), and LYQQ (**f** and **i**). Bars = 500 μm. ep, epidermis; co, cortex; ca, cambium; xy, xylem; pi, pith

Under natural conditions, the trees of MZ, QQ and LYQQ can grow upward uniformly (Additional file 1: Fig. S1). The leaves of MZ and QQ are flat, while those of LYQQ are folded inwards. In the MZ plant, the stems grew straight up, exhibiting normal shoot morphology; however, the shoots of both QQ and LYQQ tended to bend at each node and elongate in a zigzag fashion (Fig. 1a-c). Additionally, plants with zigzag-shaped shoots had shorter stems and fewer leaves than erect plants (Additional file 1: Fig. S2a), and the internode distance (between two nodes) in plants with zigzag-shaped shoots was significantly shorter than that in erect plants (Additional file 1: Fig. S2b). To precisely investigate the differences in zigzag-shaped stems at the ultrastructure level, we longitudinally dissected the stems of the QQ, LYQQ and MZ tea plants. Observation of the stem longitudinal sections showed that the tissues were basically normal, but the cell arrangement and shape differed between zigzag-shaped and erect stems (Fig. 1d-f). In QQ and LYQQ, the cortex cells tended to be disordered and arranged loosely, and the cells in both the cortex and pith exhibited aberrant shapes (Fig. 1g-i).

### RNA sequencing, reference genome alignment and new gene annotation

To investigate the regulation of zigzag-shaped stem formation in tea plants at the transcriptional level, we utilized RNA-Seq technology to analyse DEGs in the stems of MZ, QQ and LYQQ plants. In total, 46.06 million clean reads were generated from nine samples, and the sequence data were deposited in the NCBI Sequence Short Read Archive (SRA accession: PRJNA559220). After removing adaptor sequences, duplicate sequences, ambiguous reads and low-quality reads, a total of 16.37, 13.28, and 15.48 million high-quality clean reads were generated for MZ, QQ and LYQQ, respectively (Additional file 2: Table S1). The average amount of clean reads per sample was 5.2 million. The Q20 values ranged from 97.39 to 98.55%, and the Q30 values ranged from 92.26 to 95.07%. All the transcripts were aligned to the reference genome, and the average proportion of samples mapped to the genome was 76.38%. The new genes were then aligned to the Nr and KEGG databases for protein functional annotation. In total, 34,248, 34,374 and 33,598 genes were identified from MZ, QQ and LYQQ, respectively, and 28,021 (82.58%), 27,441 (80.87%) and 27,982 (82.46%) genes were annotated as known genes in MZ, QQ and LYQQ, respectively (Additional file 2: Table S2). These results indicated that the obtained high-quality transcriptomic data could be used for further analysis.

### Validation of differential expression data

**Fig. 2:**
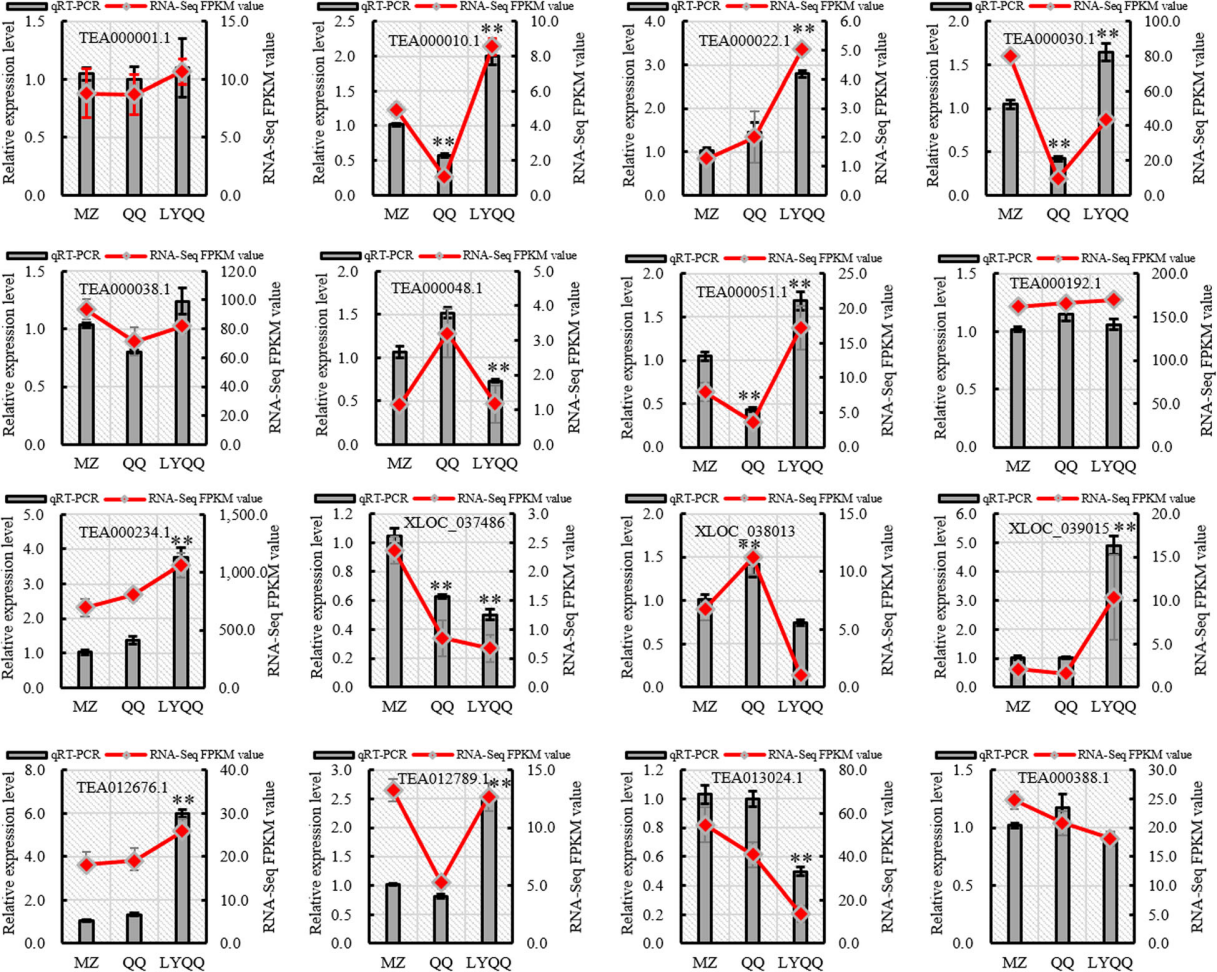
Validation of the tea plant transcriptome by qRT-PCR. Sixteen genes were selected for qRT-PCR validation, and the results are represented as the means (±SDs) of three replicates with *CsPTB1* as a control. The bars represent the qRT-PCR results, while the line represents the RNA-seq results. ** indicates a significant difference at the 0.01 level in the qRT-PCR results

To validate the reliability of the RNA-Seq results, 16 DEGs were randomly selected from the RNA-Seq data and examined using qRT-PCR. The qRT-PCR data exhibited similar expression patterns to the RNA-Seq data among the cultivars (Fig. 2), suggesting that our transcriptomic data are reliable and valid.

### Identification of DEGs and pathways in cultivar comparisons

**Fig. 3:**
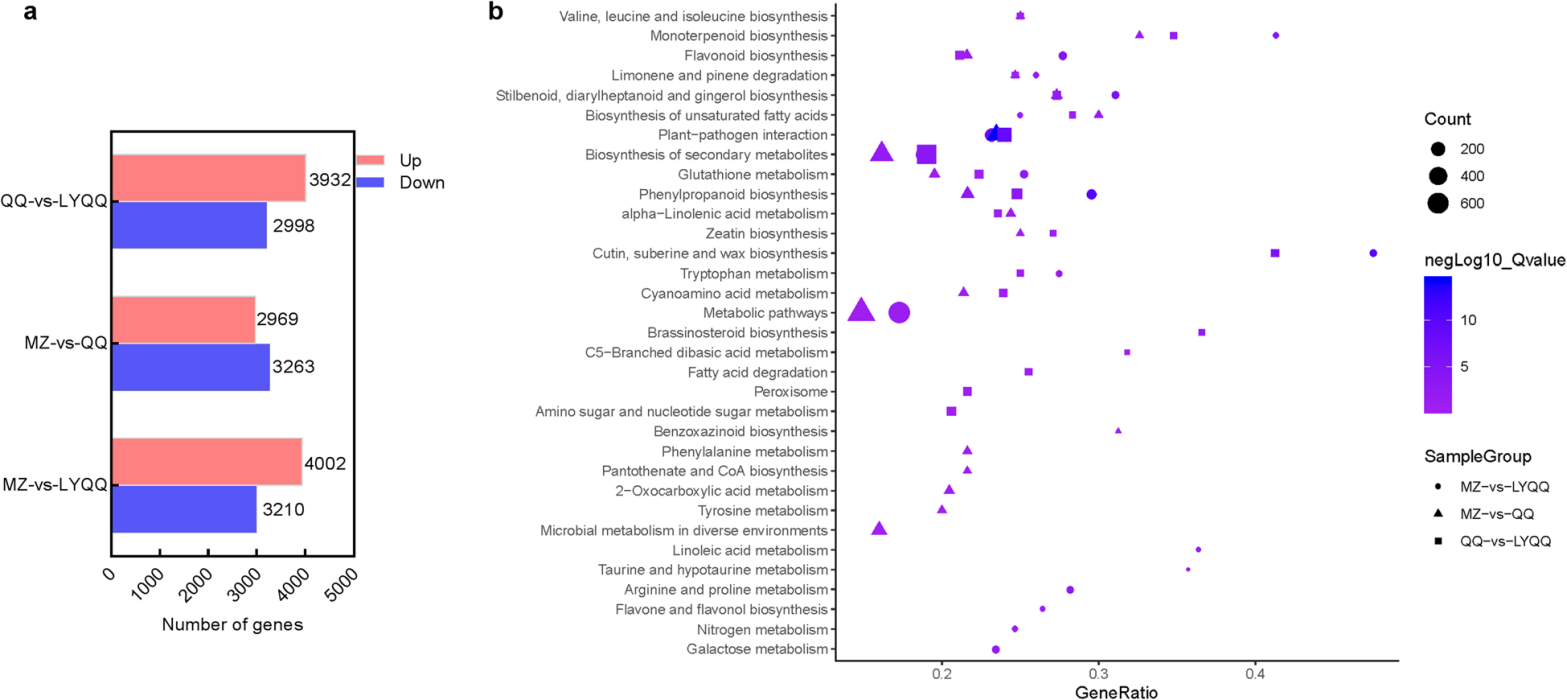
Summary of differentially expressed genes (DEGs) and their KEGG pathway enrichment analysis among the MZ, QQ, and LYQQ tea plants. **a** Number of up- and downregulated DEGs in MZ-vs-QQ, MZ-vs-LYQQ, and QQ-vs-LYQQ. **b** KEGG classification analysis of DEGs identified from MZ-vs-QQ, MZ-vs-LYQQ, and QQ-vs-LYQQ

The DEGs in each cultivar pair were then determined according to the parameters *p* value≤0.01 and |log2FC| ≥ 1. In total, 6232 DEGs, including 2969 upregulated and 3263 downregulated DEGs, were detected in MZ-vs-QQ (Fig. 3a). GO enrichment analyses showed that most of the DEGs were enriched in the terms ‘catalytic activity’, ‘metabolic process’, ‘cellular process’, ‘binding’, ‘single-organism process’, and ‘membrane’ (Additional file 1: Fig. S3). The DEGs were also subjected to KEGG pathway enrichment analyses, which showed that the pathways ‘Biosynthesis of secondary metabolites’, ‘Plant-pathogen interaction’, ‘Phenylpropanoid biosynthesis’, ‘Stilbenoid, diarylheptanoid and gingerol biosynthesis’, ‘Monoterpenoid biosynthesis’, ‘Biosynthesis of unsaturated fatty acids’, ‘alpha-Linolenic acid metabolism’, and ‘Flavonoid biosynthesis’ were significantly enriched. Additionally, we found that the zeatin biosynthesis pathway was enriched (Fig. 3b). In the MZ-vs-LYQQ comparison, a relatively high number of DEGs (7212), including 4002 upregulated and 3210 downregulated DEGs, were identified (Fig. 3a). All the DEGs could be mapped to 132 KEGG pathways, and the pathways ‘Phenylpropanoid biosynthesis’, ‘Cutin, suberine and wax biosynthesis’, ‘Plant-pathogen interaction’, ‘Stilbenoid, diarylheptanoid and gingerol biosynthesis’, ‘Flavonoid biosynthesis’, ‘Biosynthesis of secondary metabolites’, ‘Monoterpenoid biosynthesis’, ‘Glutathione metabolism’, and ‘Arginine and proline metabolism’ were significantly enriched (Fig. 3b). A total of 6930 DEGs, including 3932 upregulated and 2998 downregulated DEGs, were detected in the QQ-vs-LYQQ comparison (Fig. 3a) and mapped to 132 pathways. The DEGs in the pathways ‘Plant-pathogen interaction’, ‘Cutin, suberine and wax biosynthesis’, ‘Biosynthesis of secondary metabolites’, ‘Phenylpropanoid biosynthesis’, ‘Stilbenoid, diarylheptanoid and gingerol biosynthesis’, ‘Brassinosteroid biosynthesis’ and ‘Monoterpenoid biosynthesis’ were significantly enriched (Fig. 3b).

### Identification of DEGs and pathways involved in zigzag-shaped stem formation in tea plants

**Fig. 4:**
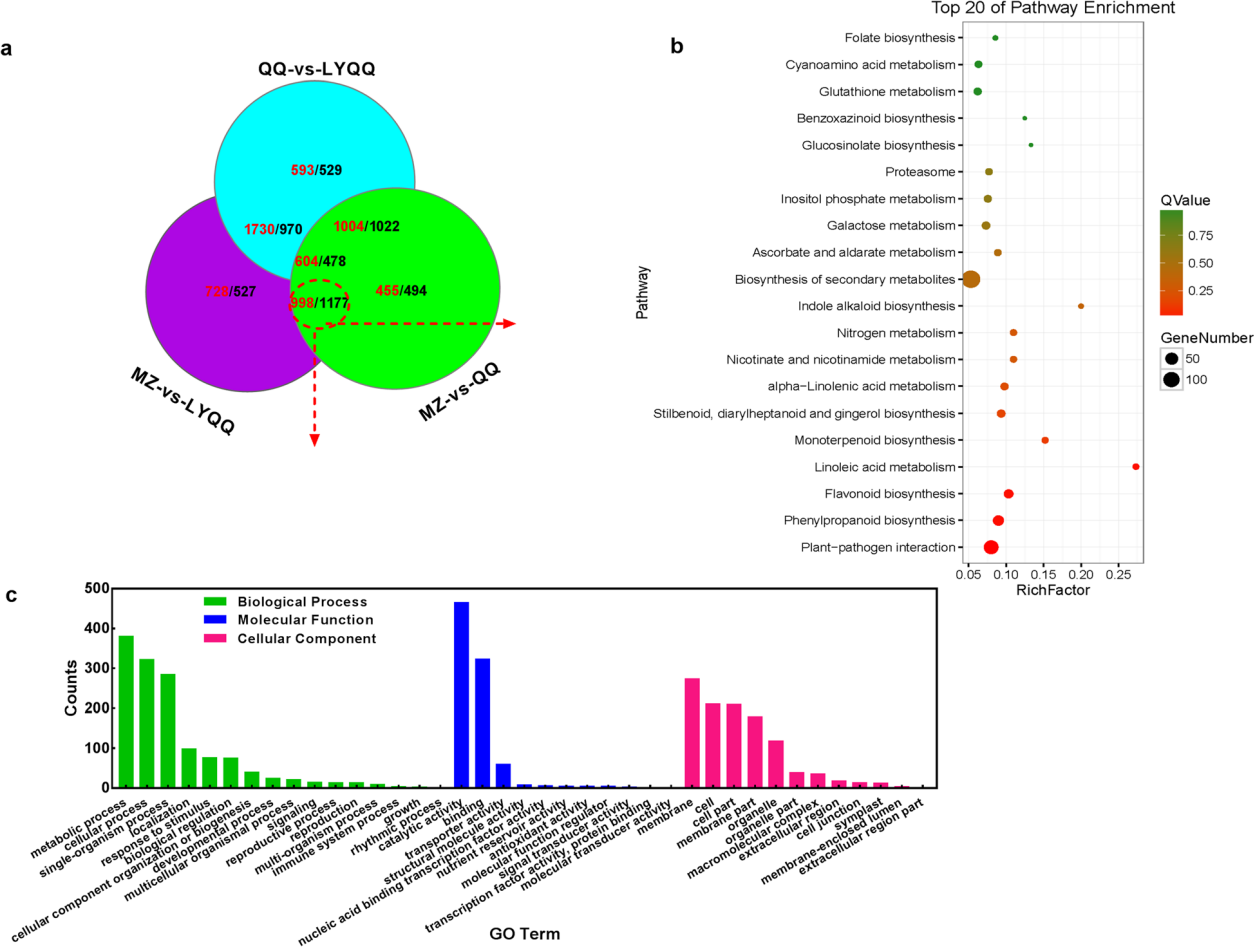
Comparative analysis of DEGs between MZ-vs-QQ and MZ-vs-LYQQ. **a** Venn diagram of the DEGs in the MZ-vs-QQ, MZ-vs-LYQQ, and QQ-vs-LYQQ comparisons. The numbers of upregulated and downregulated DEGs are indicated with red and black, respectively. **b** KEGG functional classification of 2175 DEGs that overlapped between MZ-vs-QQ and MZ-vs-LYQQ. **c** GO enrichment analysis of the 2175 DEGs identified as overlapping between MZ-vs-QQ and MZ-vs-LYQQ

We generated a Venn diagram to compare the different cultivars and showed that 1082 DEGs overlapped among the MZ-vs-LYQQ, MZ-vs-QQ, and QQ-vs-LYQQ comparisons (Fig. 4a). These DEGs were significantly enriched in the pathways of “Plant-pathogen interaction”, “Stilbenoid, diarylheptanoid and gingerol biosynthesis”, “Phenylalanine metabolism”, and “Tryptophan metabolism” (Additional file 1: Fig. S4a). In addition, a total of 1255 DEGs, including 527 downregulated and 728 upregulated DEGs, were specifically detected in the MZ-vs-LYQQ comparison (Fig. 4a). Among the top 20 pathways, “Cysteine and methionine metabolism”, “Cutin, suberine and wax biosynthesis”, “Taurine and hypotaurine metabolism”, and “Other types of O-glycan biosynthesis” were markedly enriched (Additional file 1: Fig. S4b). Unexpectedly, the number of DEGs in the MZ-vs-QQ set (949, including 494 downregulated and 455 upregulated) was lower than that in MZ-vs-LYQQ (Fig. 4a), and the pathways “Glycosphingolipid biosynthesis − globo series” and “Limonene and pinene degradation” were significantly enriched (Additional file 1: Fig. S4c). Additionally, a total of 1122 DEGs, including 593 upregulated and 529 downregulated DEGs, were specifically expressed in QQ-vs-LYQQ (Fig. 4a), but only the “Plant-pathogen interaction” pathway was significantly enriched (Additional file 1: Fig. S4d). Moreover, a total of 2175 DEGs, including 1177 downregulated and 998 upregulated DEGs, overlapped between MZ-vs-LYQQ and MZ-vs-QQ specifically, indicating that these DEGs might be associated with zigzag-shaped stem formation in both QQ and LYQQ. KEGG analysis showed that these DEGs were mainly involved in the “Plant-pathogen interaction”, “Phenylpropanoid biosynthesis”, “Flavonoid biosynthesis” and “Linoleic acid metabolism” pathways (Fig. 4b). GO enrichment analysis showed that these DEGs were significantly enriched in 59 GO terms, of which the most highly enriched components were categorized as catalytic activity (465), metabolic process (381), binding (323), cellular process (322), single-organism process (285) and membrane (274) (Fig. 4c).

### Identification of key DEGs regulating zigzag-shaped stem formation

**Fig. 5:**
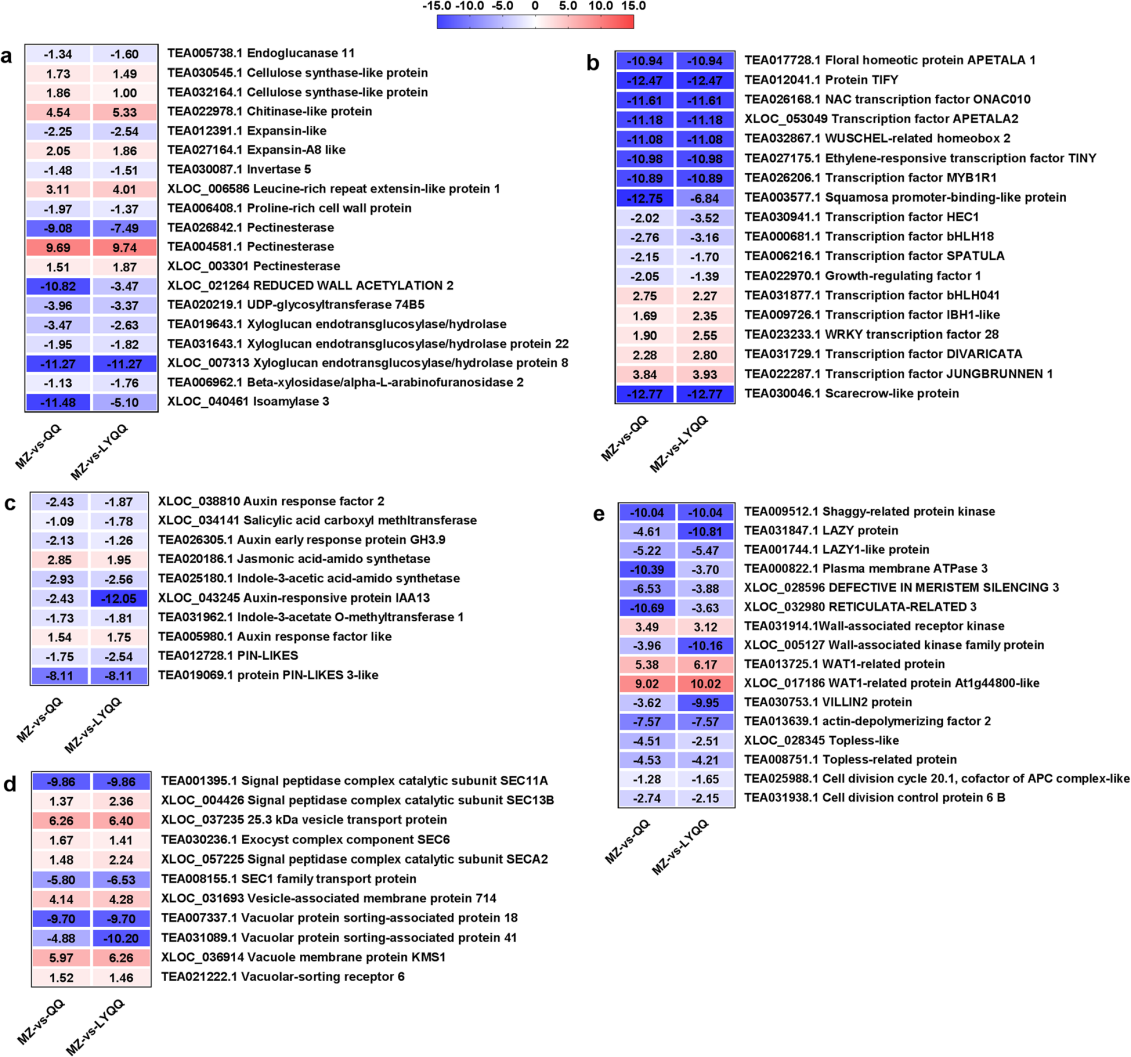
Seventy-six key DEGs identified as involved in zigzag-shaped stem formation in tea plants. DEGs associated with cell wall synthesis and cell expansion (**a**), transmembrane factors (**b**), phytohormones (**c**), vesicular trafficking (**d**), and other plant growth and development genes (**e**) were identified and analysed. The values of log2-fold changes in MZ-vs-QQ and MZ-vs-LYQQ were visualized using heat maps. Detailed information is also listed in the electronic supplementary material (Additional file 3: Table S3)

Based on the changes in expression in the comparisons MZ-vs-QQ and MZ-vs-LYQQ, 76 DEGs potentially involved in zigzag-shaped stem formation were identified (Fig. 5). Among these DEGs, 19 were associated with cell wall synthesis and cell expansion, of which seven, namely, cellulose synthase (TEA032164.1, TEA030545.1), expansin (TEA027164.1), leucine-rich repeat extensin-like protein 1 (XLOC_003301), chitinase-like protein (TEA022978.1) and pectinesterase (XLOC_003301, and TEA004581.1), were upregulated, whereas 12, especially xyloglucan endotransglucosylase/hydrolase (XLOC_007313, TEA019643.1, TEA031643.1), pectinesterase (TEA026842.1), reduced wall acetylation 2 (XLOC_021264), expansin (TEA012391.1), UDP-glycosyltransferase 74B5 (TEA020219.1) and isoamylase 3 (XLOC_040461), were downregulated in both QQ and LYQQ (Fig. 5a and Additional file 3: Table S3). Eighteen transcription factor genes, including the 13 downregulated genes floral homeotic protein APETALA 1 (TEA017728.1), TIFY (TEA012041.1), NAC transcription factor 010 (TEA026168.1), transcription factor APETALA2 (XLOC_053049), WUSCHEL-related homeobox 2 (TEA032867.1), ethylene-responsive transcription factor TINY (TEA027175.1), transcription factor MYB1R1 (TEA026206.1), squamosa promoter-binding-like protein (TEA003577.1), transcription factor HEC1 (TEA030941.1), transcription factor bHLH18 (TEA000681.1), transcription factor SPATULA (TEA006216.1), growth-regulating factor 1 (TEA022970.1), and Scarecrow-like protein (TEA030046.1) and the five upregulated genes transcription factor bHLH041 (TEA031877.1), transcription factor IBH1-like (TEA009726.1), WRKY transcription factor 28 (TEA023233.1), transcription factor DIVARICATA (TEA031729.1) and transcription factor JUNGBRUNNEN 1 (TEA022287.1), were identified (Fig. 5b and Additional file 3: Table S3). In addition, 10 DEGs involved in auxin, jasmonic acid, and salicylic acid metabolism and transport were also identified in the list of key DEGs; interestingly, except for jasmonic acid-amido synthetase (TEA020186.1), the remaining genes, especially PIN3 (TEA019069.1), were downregulated in both QQ and LYQQ (Fig. 5c and Additional file 3: Table S3). Furthermore, seven DEGs involved in protein processing and transportation on the endoplasmic reticulum and vesicles, namely, vesicle-associated membrane protein 714 (XLOC_031693), SEC1 family transport protein, signal peptidase complex catalytic subunit SEC11A (TEA001395.1), SEC13B (XLOC_004426), SECA2 (XLOC_057225), SEC6 (TEA030236.1), SEC11A (TEA001395.1), and SEC22 (XLOC_037235); the three vacuolar protein sorting-related proteins VPS18 (TEA007337.1), VPS41 (TEA031089.1) and VSR6 (TEA021222.1); and the vacuole membrane protein KMS1 (XLOC_036914) were identified from the MZ-vs-QQ and MZ-vs-LYQQ comparisons (Fig. 5d and Additional file 3: Table S3). Among these DEGs, VPS18, VPS41, SEC11A and SEC1 were significantly repressed in both QQ and LYQQ. Genes that regulate cell division (cell division cycle 20.1 and cell division control protein 6 B) and plant development, such as shaggy-related protein kinase, DEFECTIVE IN MERISTEM SILENCING 3 (XLOC_028596), RETICULATA-RELATED 3 (XLOC_032980), TOPLESS-like (XLOC_028345), TOPLESS-related protein (TEA008751.1), LAZY protein (TEA031847.1) and LAZY 1-like (TEA001744.1), were also identified, and all of these genes were downregulated in both QQ and LYQQ (Fig. 5e and Additional file 3: Table S3). Moreover, the DEGs VILLIN2 protein (*VLN2*) and actin-depolymerizing factor 2 (*ADF2*) were suppressed in both QQ and LYQQ (Fig. 5e and Additional file 3: Table S3).

### Metabolic analysis and key metabolite identification

**Fig. 6:**
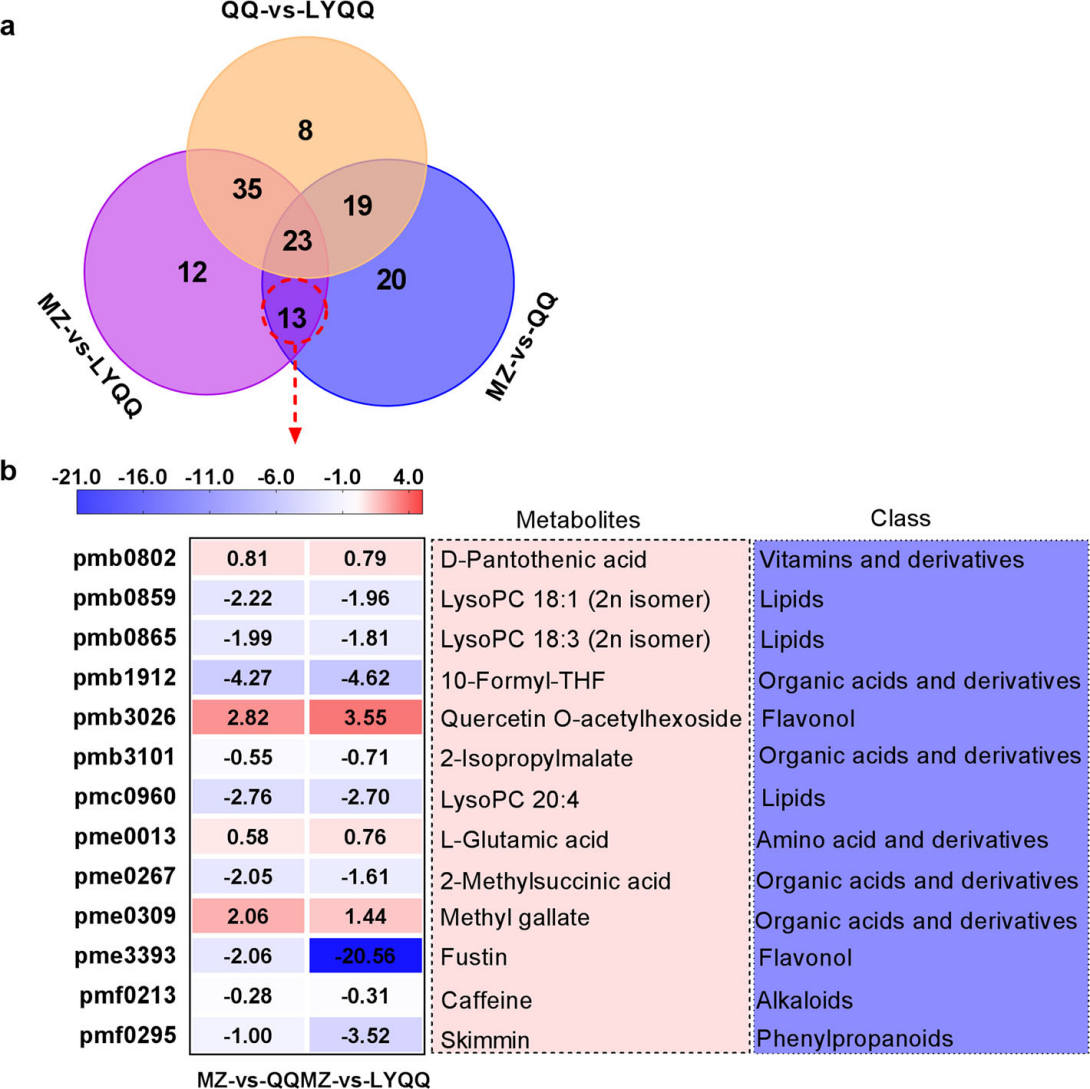
Identification of differential metabolites involved in zigzag-shaped stem regulation in tea plants. **a** Venn diagram analysis of differential metabolites in the MZ-vs-QQ, MZ-vs-LYQQ, and QQ-vs-LYQQ comparisons. **b** Thirteen differential metabolites identified as overlapping between MZ-vs-QQ and MZ-vs-LYQQ

To investigate the metabolic pathways involved in zigzag-shaped stem formation, the metabolites in the stems of QQ, LYQQ and MZ were detected using UPLC-ESI-TOF-MS/MS. In total, 752 metabolites clustered into 97 KEGG pathways were identified from QQ, LYQQ and MZ, and among these metabolites, 75, 84 and 86 metabolites showed significantly different levels in the MZ-vs-QQ, MZ-vs-LYQQ and QQ-vs-LYQQ comparisons, respectively (Additional file 1: Fig. S5). The Venn diagram analysis showed that 13 metabolites overlapped between MZ-vs-QQ and MZ-vs-LYQQ, which were our metabolites of interest (Fig. 6a), and the results indicated that these differential metabolites might be associated with zigzag-shaped stem formation in tea plants. Based on their log2 fold change values, these differential metabolites were visualized as a heat map in Fig. 6b. Quercetin O-acetylhexoside, methyl gallate, D-pantothenic acid and L-glutamic acid were upregulated in both QQ and LYQQ, whereas the remaining metabolites, including fustin, 10-formyl-THF, skimmin, LysoPC 20:4, LysoPC 18:1 (2n isomer), LysoPC 18:3 (2n isomer), 2-methylsuccinic acid, 2-isopropylmalate, and caffeine, were significantly downregulated in tea plants with zigzag-shaped shoots.

## Discussion

Plants are sessile and cannot move freely from their habitat, even if they encounter adverse environments. Generally, plant shoots grow upward, whereas roots grow downward; therefore, shoot architecture is a determinant of plant morphology. In tea plants, the shoots play a substantial role in determining the horticultural characteristics and tea yield, and almost all tea plant cultivars exhibit straight stems. The two tea plants QQ and LYQQ, with zigzag-shaped shoots, were identified and planted previously; however, with advances in research, there is increasing interest in the mechanism by which QQ and LYQQ develop their zigzag-shaped shoots. Zigzag-shaped shoots, which occasionally appear in garden plants, are an unusual trait in woody plants, but there is relatively little information regarding this feature. Similarly, in *Arabidopsis*, *zig* mutant stems elongate in a zigzag fashion, curve upward at the internodes and, interestingly, exhibit abnormal gravitropism in hypocotyls and shoots [8–11].

To understand the cause of this anomalous zigzag-shaped morphology, we observed stem sections using optical microscopy. There were no aberrations in the main tissue structures (Fig. 1). However, differences in cell arrangement and shape were observed between the zigzag-shaped stems of QQ and LYQQ and the MZ stems, including the arrangement of cortex cells, which were disordered and loosely arranged, and the cells in the cortex and pith exhibited aberrant shapes (Fig. 1). Consistent with our findings, similar cell shapes and arrangements were found in the *zig-1* mutant stems of *Arabidopsis* [8]; however, different cell shapes and arrangements were observed in the epidermal layer and pith of *Arabidopsis* than in the cortex and pith of tea plants; this may represent a difference between woody plants and Arabidopsis. Thus, zigzag-shaped stems might be caused by the anomalous shape, arrangement and expansion of cells in tea plants.

To investigate the molecular mechanism of zigzag-shaped stem formation in tea plants, shoots were collected, and transcriptome sequence analysis was performed. In total, 46.06 million clean reads were generated, and 6232, 7212 and 6930 DEGs were identified from MZ-vs-QQ, MZ-vs-LYQQ and QQ-vs-LYQQ, respectively (Additional file 2: Table S1 and Fig. 3b). These DEGs were mainly enriched in several pathways, such as ‘Biosynthesis of secondary metabolites’, ‘Plant-pathogen interaction’, and ‘Stilbenoid, diarylheptanoid and gingerol biosynthesis’, indicating that these pathways might be associated with differences among cultivars (Fig. 3b). To gain insights into the DEGs that specifically regulate zigzag-shaped stem development, we made a Venn diagram of cultivar comparisons and identified 2175 overlapping DEGs between MZ-vs-QQ and MZ-vs-LYQQ, which were mainly enriched in the “Plant-pathogen interaction”, “Phenylpropanoid biosynthesis”, “Flavonoid biosynthesis” and “Linoleic acid metabolism” pathways (Fig. 4). Among these pathways, the “Phenylpropanoid biosynthesis” pathway serves as a source of metabolites in plants, is involved in the biosynthesis of lignin, flavonoids, coumarins and lignans, and plays a fundamental role in plant structural support [29, 30]. It has been found that, in *Arabidopsis*, mutation of the genes encoding cinnamate 4-hydroxylase (C4H) and hydroxycinnamoyl-coenzyme A shikimate:quinate hydroxycinnamoyl-transferase (HCT) involved in this pathway resulted in changes in the structural, developmental, and reproductive phenotypes of *Arabidopsis* plants [31–33]. Interestingly, the flavonols quercetin and kaempferol have been shown to inhibit polar auxin transport and to enhance consequent localized auxin accumulation [34–36]. Recently, Kuhn, et al. [34] suggested that flavonols could modulate auxin transport by modifying the antagonistic kinase/phosphatase equilibrium. Therefore, flavonols could affect auxin transport in zigzag-shaped shoots. On the other hand, Ramos, et al. [37] found that the concentration of quercetin in the upper half of longitudinally dissected 45-degree-inclined radiata pine seedlings was higher than that in the lower half and than that in non-inclined seedlings when seedlings were inclined for 1 month, indicating that quercetin can accumulate in inclined shoots such as the internodes of zigzag-shaped shoots. Consistently, we observed that a *C4H* gene (TEA016772.1) was significantly differentially expressed between MZ-vs-QQ and MZ-vs-LYQQ; moreover, we found that skimmin (pmf0295) expression was reduced in the MZ-vs-QQ (log2 FC: − 0.995) and MZ-vs-LYQQ (log2 FC: − 3.52) comparisons. Therefore, the zigzag-shaped stems of tea plants might be partially related to flavonoids, especially flavonol-mediated auxin transport.

It is well recognized that a zigzag-shaped inflorescence stem results from the mutation of *zig* (*zigzag*)/*sgr4*, which encodes VPS10 interacting 11 (VTI11), a Qb-SNARE involved in vesicle transport between the trans-Golgi network and vacuole that causes abnormal gravitropism in *Arabidopsis* when mutated [8, 10, 11, 38, 39]. SNAREs play an important role in membrane fusion at the vacuole and are involved in the regulation of amyloplast sedimentation in response to gravity and in cell shape development [11, 39]. ZIG can form a complex with other types of SNARE proteins, including SYP22/SGR3/VAM3, SYP5, and VAMP727, most likely at the prevacuolar compartment and vacuole [11, 40–42]. Although *ZIG* and the other *SGR* genes were not significantly expressed, 11 genes mainly involved in vesicular trafficking were identified (Fig. 5d and Additional file 3: Table S3). Among these genes, both vacuolar protein sorting-associated protein genes (*VPS18* and *VPS41*) were repressed in QQ and LYQQ, whereas the expression levels of vesicle-associated membrane protein 714 (*VAMP714*) and vacuolar-sorting receptor 6 (*VSR6*) were increased in QQ and LYQQ. It has been established that the phenotype of *zig-1* could be partially suppressed by mutations in the *zig suppressor1* (*ZIP1*), *ZIP2*, *ZIP3* and *ZIP4* genes in *Arabidopsis* [38, 43, 44]. Niihama, et al. [44] reported that *ZIP2*, which encodes an AtVPS41/AtVAM2 protein, is involved in protein sorting to the vacuole in *Arabidopsis*, and the *zip2* mutation is a missense mutation. These results indicate that zigzag-shaped stem formation is mainly related to abnormal gravitropism responses mediated by membrane trafficking. Additionally, we also found that six SEC family genes were significantly expressed in the MZ-vs-QQ and MZ-vs-LYQQ comparisons (Fig. 5d and Additional file 3: Table S3); these genes are crucial for SNARE complex assembly and preprotein translocation [45, 46]. Interestingly, we found that both *LAZY* genes (TEA031847.1, TEA001744.1) were markedly repressed in QQ and LYQQ (Fig. 5d and Additional file 3: Table S3). It is well known that loss-of-function mutation of *LAZY1* enhances polar indole-3-acetic acid (IAA) transport and reduces shoot gravitropism and therefore regulates the growth angle of lateral branches in rice, *Arabidopsis* and maize [17–19, 21, 47]; however, the function of *LAZY* in woody plants remains to be elucidated. Therefore, we propose that the development of zigzag-shaped stems in tea plants might be associated with a change in the shoot gravitropism response, especially one affecting the disruption of membrane trafficking to vacuoles, although the *ZIG*/*SGR4* gene did not exhibit a significant change in expression.

The plant hormone auxin is important for organ growth and cell morphogenesis. In this study, seven DEGs involved in auxin metabolism, transport and signalling were identified by comparison of the MZ-vs-QQ and MZ-vs-LYQQ sets, and all of the genes were downregulated in QQ and LYQQ (Fig. 5c and Additional file 3: Table S3). Numerous studies have suggested that polar auxin transport, which is primarily determined by polar localization of PIN auxin efflux carriers, controls plant tropism in roots and shoots and plays essential roles in plant growth [13, 48, 49]. Gerttula, et al. [50] proposed that *PIN* expression in different cambium cells results in auxin transport towards the cambium in the top and bottom of the stem to trigger wood formation in response to gravity in woody stems. Consistent with this finding, we found that the expression of *PIN* DEGs was repressed in QQ and LYQQ (Fig. 5c and Additional file 3: Table S3); these changes in expression in QQ and LYQQ might alter the polar transport of auxin and then affect the auxin gradients between stem sides; therefore, the shoots exhibit bending. Wu, et al. [51] reported that rice *VLN2*, a type of actin-binding protein involved in microfilament regulation, affects the recycling of PIN2 and polar auxin transport, and *vln2*-defective mutant plants exhibited a hypersensitive gravitropic response and twisted roots and shoots at the seedling stage. In this study, we also found that *VLN2* (TEA030753.1) was markedly repressed in QQ and LYQQ (Fig. 5e and Additional file 3: Table S3), suggesting that zigzag-shaped stems in tea plants might be related to polar auxin transport and the gravitropism response.

Moreover, cell expansion might exert a compressive force, leading to bending of the stem. Our results showed that genes involved in cell expansion and cell wall synthesis, such as expansin, REDUCED WALL ACETYLATION, and xyloglucan endotransglucosylase/hydrolase protein, were differentially expressed (Fig. 5a and Additional file 3: Table S3), resulting in alteration of cell expansion and elongation. In addition, *wall-associated kinase* (WAK) genes were also differentially expressed in MZ-vs-QQ and MZ-vs-LYQQ, suggesting that WAK-mediated cell expansion and signalling pathways might be required for zigzag-shaped stem formation in tea plants. It is possible that cell expansion in the stems can produce a force that can lead to zigzag-shaped shoots. Importantly, cell expansion and differentiation predominantly rely on auxin [12, 52, 53]; thus, the mechanism of auxin regulation in zigzag-shaped shoot development needs to be studied precisely in the different tissue sides of zigzag-shaped stems.

In this study, 20 transcription factors (TFs) belonging to different TF families, including two ARFs, were differentially expressed in MZ-vs-QQ and MZ-vs-LYQQ, and most of these TFs were significantly repressed in QQ and LYQQ (Fig. 5b and Additional file 3: Table S3). Almost all of these genes had homologues associated with plant growth and developmental regulation in other plants. For instance, DIVARICATA (TEA031729.1), a MYB-type TF, could interact with WOX to control wood formation in poplar [54]; overexpression of *IBH1* causes erect leaves in rice and dwarfism in *Arabidopsis* [55]; and HEC1 coordinates with WUS to promote stem cell proliferation in the shoot meristem [56, 57]. In *Paulownia kawakamii*, antisense suppression of *PkMADS1*, a regulator of shoot morphogenesis, resulted in zigzag-shaped shoots [58]. Interestingly, in some transformants of antisense *PkMADS1*, the main shoot apex appeared to be used up early during leaf formation, and then, the axillary bud of the youngest leaf took over the function of the apical meristem, resulting in the formation of one leaf per node and the zigzag-shaped growth habit for the stem, indicating that central and lateral meristem fates regulated by a variety of TFs may relate to zigzag-shaped shoot formation. Recently, several genes that regulate architectural phenotypes in woody plants, such as ARBORKNOX 2 (*ARK2*) in Populus [50] and GA INSENSITIVE DWARF 1C (*GID1C*), Tiller Angle Control 1 (*TAC1*) and *WEEP* in peach [23, 59, 60], have been isolated; however, most of these genes are related to the plant gravitropic response, and none of these genes encode TFs. Additionally, among the nine *SGR* genes, *SGR1* and *SGR7* encode scarecrow (SCR) and GRAS family TFs, respectively. We found that the *SCR* gene (TEA030046.1) was markedly repressed in QQ and LYQQ (Fig. 5b and Additional file 3: Table S3). Plant growth and development are directed by a TF-mediated complex network integrated with plant hormones, enzymes and other cellular components; therefore, as-yet-unknown TFs may be involved in the regulation of plant architectural phenotypes.

## Conclusion

In the current study, we investigated the mechanism of zigzag-shaped shoot formation in tea plants using comparative transcriptomics and metabolomic analysis. The results showed that zigzag-shaped stem development in tea plants might be regulated by a complex network involving vesicular trafficking, phytohormones, cell expansion, secondary metabolism, and diverse transcription factors. Importantly, zigzag-shaped shoot formation might be closely related to alterations in the gravitropic response and polar auxin transport in tea plants. Our findings provide insights into zigzag-shaped shoot formation in tea plants and serve as a valuable foundation for further investigations of architecture formation in woody plants.

## Methods

### Plant materials

The tender stems (between the first and third leaves from the apical bud) of tea plants with erect (*C. sinensis* var. Meizhan, MZ) and zigzag-shaped (*C. sinensis* var. Qiqu, QQ; *C. sinensis* var. Lianyuanqiqu, LYQQ) shoots were collected from the germplasm resource garden of Wuyi University, Wuyishan City, Fujian Province, China, in October 2018. A portion of each sample was immediately frozen in liquid nitrogen and then stored at − 80 °C until use for transcriptome sequencing and metabolite analysis. The remaining samples were fixed in cold fixative solution (4 °C) for section observation. All samples were examined in triplicate and used as biological replicates.

### Tissue section observation

Longitudinal stem sections were sliced and stained as previously described by Ile, et al. [61] with minor modifications. At least seven stems from each tea plant cultivar were cut into fragments (< 0.5 cm) and fixed in fixative solution over 72 h at 4 °C. The stems were dehydrated by transferring them sequentially through a series of increasing concentrations of ethanol (75 to 100% absolute ethanol), followed by 100% ethanol, ethanol:xylene (2:1), ethanol:xylene (1:1), ethanol:xylene (1:2), pure xylene, pure xylene (each for 1 h), xylene: Paraplast (1:1) for 24 h, and molten Paraplast (melting point 65 °C) for 48 h and finally embedded in freshly molten Paraplast. Central longitudinal sections of 5 μm thickness per stem were cut using a disposable-blade rotary microtome (RM2016, Leica, Shanghai, China), allowed to stand overnight and dried at 40 °C for 4 h. Sections were dewaxed in xylene, hydrated gradually in decreasing concentrations of ethanol (from 100 to 70%) and stained with safranine (1.0% for 2.5 h) followed by Fast Green (1.0% for 8 s) in ethanol. After staining, the stem sections were observed under a Leica DMi8 inverted microscope (Leica, Shanghai, China).

### UPLC-ESI-MS/MS analysis and differential metabolite identification

A total of 100 mg of powder from crushed freeze-dried samples was weighed and extracted overnight at 4 °C with 1.0 ml of 70% aqueous methanol containing 0.1 mg/L lidocaine as an internal standard. Following centrifugation at 10,000×g for 10 min, the supernatant was absorbed and filtered (SCAA-104, 0.22-μm pore size; ANPEL, Shanghai, China) before liquid chromatography-tandem mass chromatography (LC-MS/MS) analysis. Quality control samples were mixed with all the samples to ensure the reproducibility of the entire experiment. The extracted compounds were analysed using an ultra performance liquid chromatography-electrospray ionization-tandem mass spectrometry (UPLC-ESI-MS/MS) system (UPLC, Shim-pack UFLC SHIMADZU CBM30A; MS/MS, Applied Biosystems 6500 QTRAP) [62]. For compounds separation, column, mobile phases, and operation parameters were chosen or set following the description by Xu et al. [63]. The effluent was further transported to an ESI-triple quadrupole-linear ion trap (QTRAP)-MS.

Analyst 1.6.1 software was employed for metabolite identification. Details on data filtering, peak detection, alignment, calculations, and differential metabolite identification were described by Tang et al. [64]. Particularly, metabolites with T-test *P* values < 0.05 and VIP ≥ 1 were considered as differential metabolites and mapped to KEGG metabolic pathways for pathway enrichment analysis (FDR ≤ 0.05) [65].

### RNA isolation, library construction, Illumina sequencing, and data processing

The total RNA from each sample, which consisted of at least 30 shoots collected from over 10 tea individuals, was extracted using the RNAprep Pure Plant Kit (TIANGEN, Beijing, China). The total RNA quantity and integrity were evaluated and estimated using an Agilent Bioanalyzer 2100 system (Agilent, Santa Clara, CA, USA) and a NanoDrop™ ultraviolet spectrophotometer (Thermo, Waltham, MA, USA), respectively. cDNA libraries were constructed using the NEBNext Ultra RNA Library Prep kit (Gene, Beijing, China) and sequenced using an Illumina HiSeq TM 2500 instrument (Genedenovo Biotechnology Co., Guangzhou, China). High-quality clean reads were acquired by removing adaptor sequences, reads containing more than 10% unknown nucleotides (N), and low-quality reads containing more than 50% low-quality (Q value≤20) bases; then, the Q20 and Q30 values, GC content, and sequence duplication levels of the clean reads were calculated. The clean reads of each sample were then mapped to the tea plant genome by TopHat2 [66] (version 2.0.3.12).

Gene abundances were quantified with RSEM software, and the unigene expression levels were quantified using fragments per kilobase of transcript per million mapped reads (FPKM) values [67]. To identify DEGs across samples or groups, the edgeR package (http://www.rproject.org/) was used, and genes with |log2FC| ≥1 and FDR < 0.05 were identified as DEGs. DEGs were then subjected to enrichment analysis of GO functions and KEGG pathways using the GOseq R package [68] and KOBAS software [69], respectively.

### Quantitative real-time PCR validation

To validate the reliability of the gene expression analysis in this study, 16 genes were selected for real-time qPCR analysis. Total RNA samples were used for cDNA synthesis according to the method recommended by the SuperScript® III Reverse Transcriptase kit manufacturer. Information about the primers used for qRT-PCR analysis is listed in Additional file 4: Table S4. qRT-PCR was performed using SYBR Premix Ex Taq™ II (TaKaRa, Dalian, China) in a CFX96 Touch real-time PCR system (BIO-RAD, California, USA) according to the manufacturer’s protocol, and amplification was performed as previously reported [70]. The results were calculated using the 2^−ΔΔCT^ method [71] with the *CsPTB1* gene as a control [72]. Each sample was examined in triplicate.

## Supplementary information

**Additional file 1: Figure S1** Growth architectures of MZ (a and d), QQ (b and e) and LYQQ (c and f) in a natural tea garden. The shoots of QQ and LYQQ exhibit a zigzag shape. **Figure S2** Stem morphology (a) and inter-node length (b) analysis of MZ, LYQQ and QQ. Mature stems were collected on February 2020. The length of the inter-node between the third and fourth nodes (red lines) was determined (*n* = 5). ** indicates a significant difference at the 0.01 level. **Figure S3** GO enrichment analysis of DEGs identified from the comparisons MZ-vs-QQ (a), MZ-vs-LYQQ (b), and QQ-vs-LYQQ (c). **Figure S4** KEGG enrichment analysis of DEGs identified from the comparisons MZ-vs-LYQQ_MZ-vs-QQ _QQ-vs-LYQQ (a), MZ-vs-LYQQ (b), MZ-vs-QQ (c) and QQ-vs-LYQQ (d). **Figure S5**. Differential metabolites identified from MZ-vs-QQ, MZ-vs-LYQQ, and QQ-vs-LYQQ.

**Additional file 2: Table S1** Summary of the RNA-Seq data derived from MZ, QQ, and LYQQ. **Table S2** Statistics of the number of detected genes in each cultivar.

**Additional file 3: Table S3** Seventy-six key DEGs identified to be involved in zigzag-shaped stem formation in tea plants.

**Additional file 4: Table S4** DEGs and primers used for qRT-PCR validation of the transcriptome.

## Data Availability

The datasets generated and analysed during the current study are available in the NCBI Sequence Read Archive (SRA), with the link of https://www.ncbi.nlm.nih.gov/bioproject/PRJNA559220, under the accession number PRJNA559220.

## Abbreviations

DEGs: Differentially expressed genes
FDR: False discovery rate
FPKM: Fragments per kilobase of transcript per million mapped reads
LC-MS/MS: Liquid chromatography-tandem mass chromatography
LYQQ: Lianyuanqiqu
MZ: Meizhan
PIN: PIN-FORMED
QQ: Qiqu
qRT-PCR: quantitative real-time PCR
SGR: Shoot gravitropism
UPLC-ESI-MS/MS: Ultra performance liquid chromatography-electrospray ionization-tandem mass spectrometry
ZIG: Zigzag
ZIP: Zig suppressor1
