# Supplementary figures and images for: Transcriptome and metabolite analyses provide insights into zigzag-shaped stem formation in tea plants (Camellia sinensis)
Source: BMC Plant Biol. 2020 Mar 4;20:98. doi: 10.1186/s12870-020-2311-z (PMC7057490; doi:10.1186/s12870-020-2311-z)

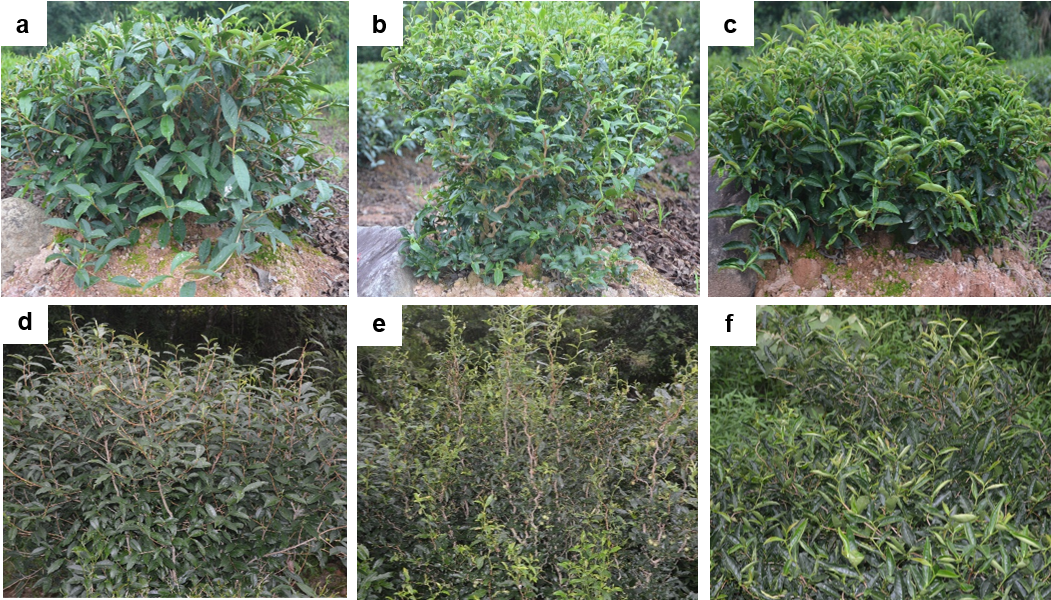

Supplement: Supplementary file 1 — Additional file 1: Figure S1 Growth architectures of MZ (a and d), QQ (b and e) and LYQQ (c and f) in a natural tea garden. The shoots of QQ and LYQQ exhibit a zigzag shape. Figure S2 Stem morphology (a) and inter-node length (b) analysis of MZ, LYQQ and QQ. Mature stems were collected on February 2020. The length of the inter-node between the third and fourth nodes (red lines) was determined (n = 5). ** indicates a significant difference at the 0.01 level. Figure S3 GO enrichment analysis of DEGs identified from the comparisons MZ-vs-QQ (a), MZ-vs-LYQQ (b), and QQ-vs-LYQQ (c). Figure S4 KEGG enrichment analysis of DEGs identified from the comparisons MZ-vs-LYQQ_MZ-vs-QQ _QQ-vs-LYQQ (a), MZ-vs-LYQQ (b), MZ-vs-QQ (c) and QQ-vs-LYQQ (d). Figure S5. Differential metabolites identified from MZ-vs-QQ, MZ-vs-LYQQ, and QQ-vs-LYQQ. [file 12870_2020_2311_MOESM1_ESM.zip › Additional file 1 Fig. S1.tif]

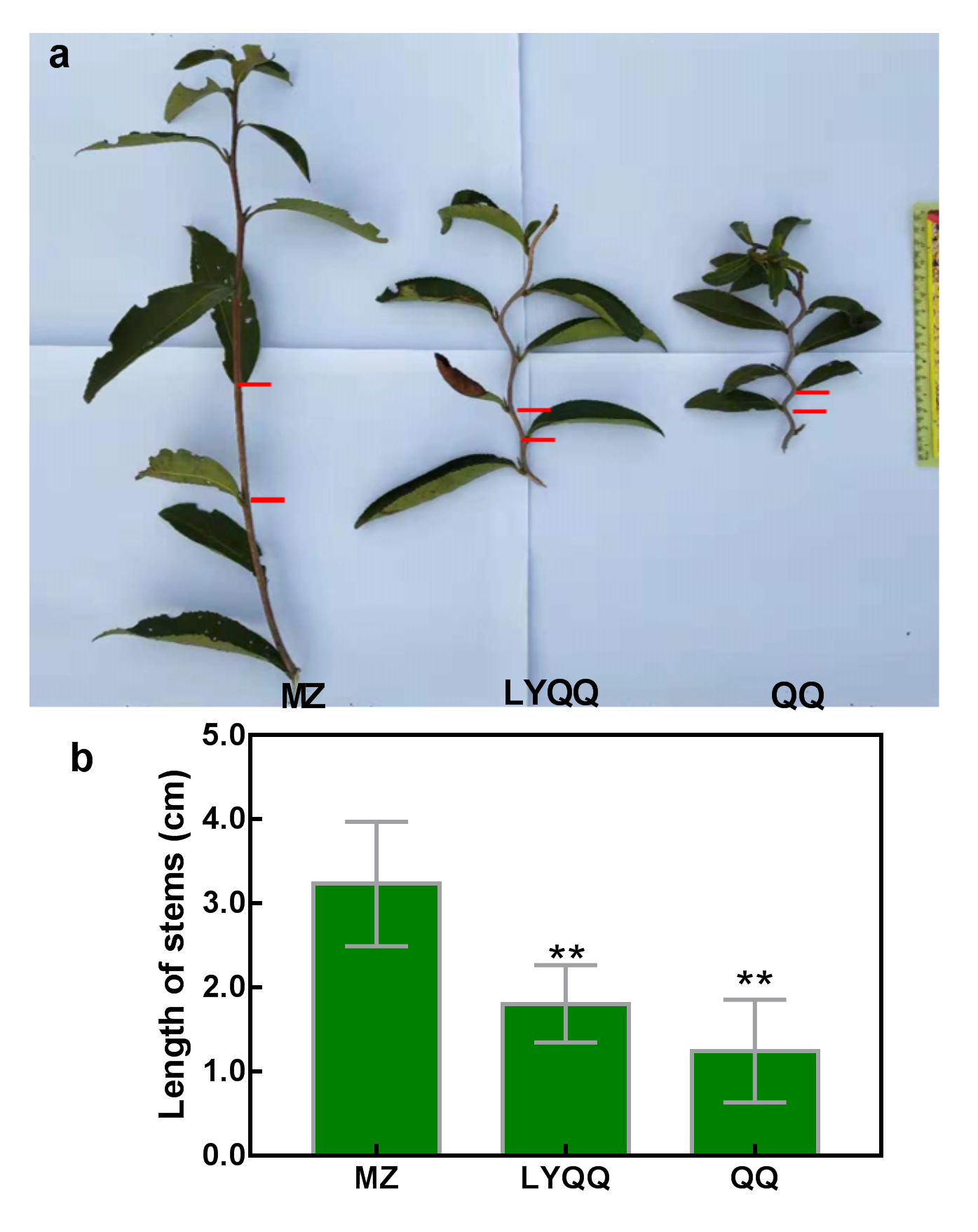

Supplement: Supplementary file 1 — Additional file 1: Figure S1 Growth architectures of MZ (a and d), QQ (b and e) and LYQQ (c and f) in a natural tea garden. The shoots of QQ and LYQQ exhibit a zigzag shape. Figure S2 Stem morphology (a) and inter-node length (b) analysis of MZ, LYQQ and QQ. Mature stems were collected on February 2020. The length of the inter-node between the third and fourth nodes (red lines) was determined (n = 5). ** indicates a significant difference at the 0.01 level. Figure S3 GO enrichment analysis of DEGs identified from the comparisons MZ-vs-QQ (a), MZ-vs-LYQQ (b), and QQ-vs-LYQQ (c). Figure S4 KEGG enrichment analysis of DEGs identified from the comparisons MZ-vs-LYQQ_MZ-vs-QQ _QQ-vs-LYQQ (a), MZ-vs-LYQQ (b), MZ-vs-QQ (c) and QQ-vs-LYQQ (d). Figure S5. Differential metabolites identified from MZ-vs-QQ, MZ-vs-LYQQ, and QQ-vs-LYQQ. [file 12870_2020_2311_MOESM1_ESM.zip › Additional file 1 Fig. S2.tif]

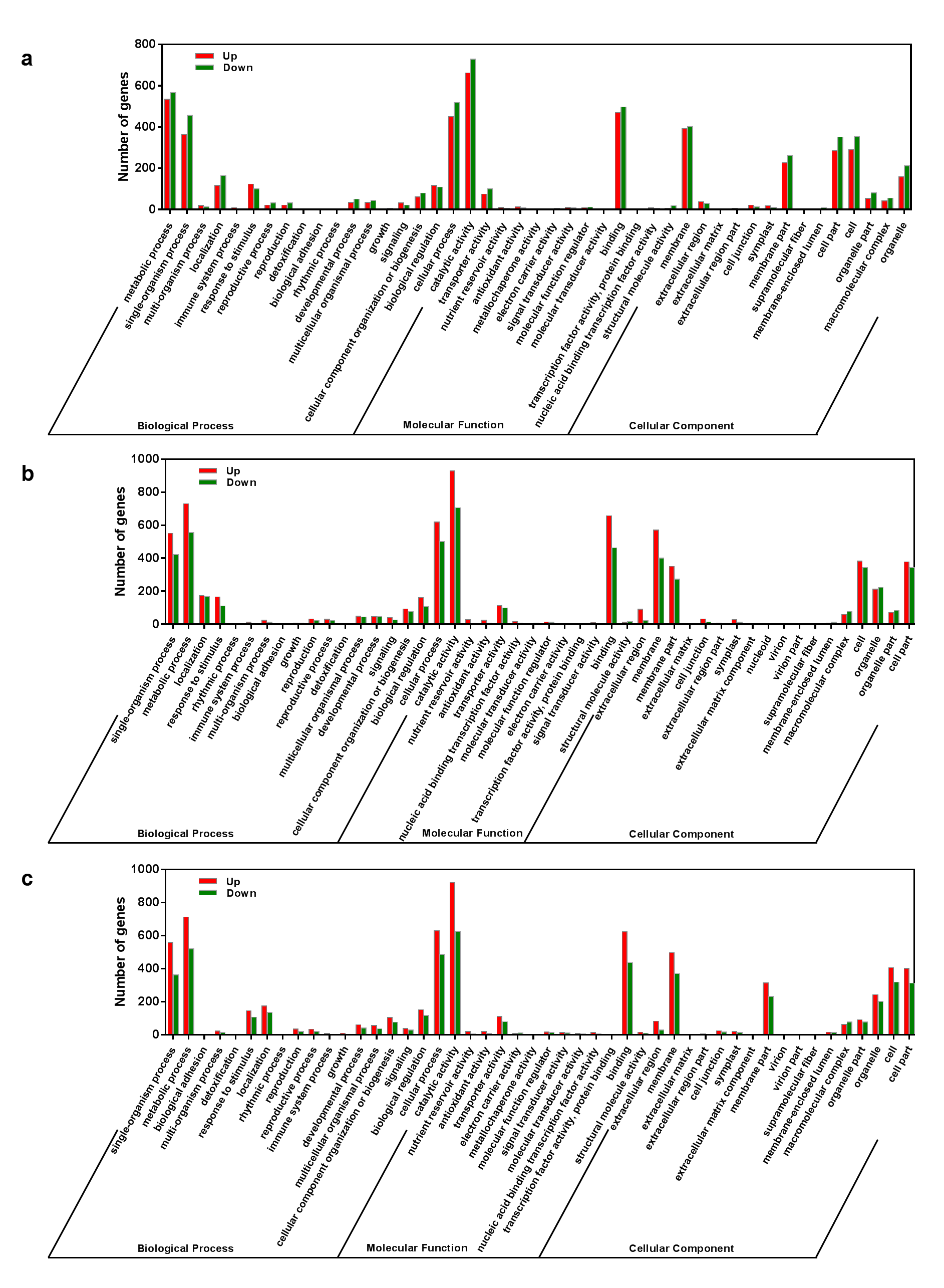

Supplement: Supplementary file 1 — Additional file 1: Figure S1 Growth architectures of MZ (a and d), QQ (b and e) and LYQQ (c and f) in a natural tea garden. The shoots of QQ and LYQQ exhibit a zigzag shape. Figure S2 Stem morphology (a) and inter-node length (b) analysis of MZ, LYQQ and QQ. Mature stems were collected on February 2020. The length of the inter-node between the third and fourth nodes (red lines) was determined (n = 5). ** indicates a significant difference at the 0.01 level. Figure S3 GO enrichment analysis of DEGs identified from the comparisons MZ-vs-QQ (a), MZ-vs-LYQQ (b), and QQ-vs-LYQQ (c). Figure S4 KEGG enrichment analysis of DEGs identified from the comparisons MZ-vs-LYQQ_MZ-vs-QQ _QQ-vs-LYQQ (a), MZ-vs-LYQQ (b), MZ-vs-QQ (c) and QQ-vs-LYQQ (d). Figure S5. Differential metabolites identified from MZ-vs-QQ, MZ-vs-LYQQ, and QQ-vs-LYQQ. [file 12870_2020_2311_MOESM1_ESM.zip › Additional file 1 Fig. S3.tif]

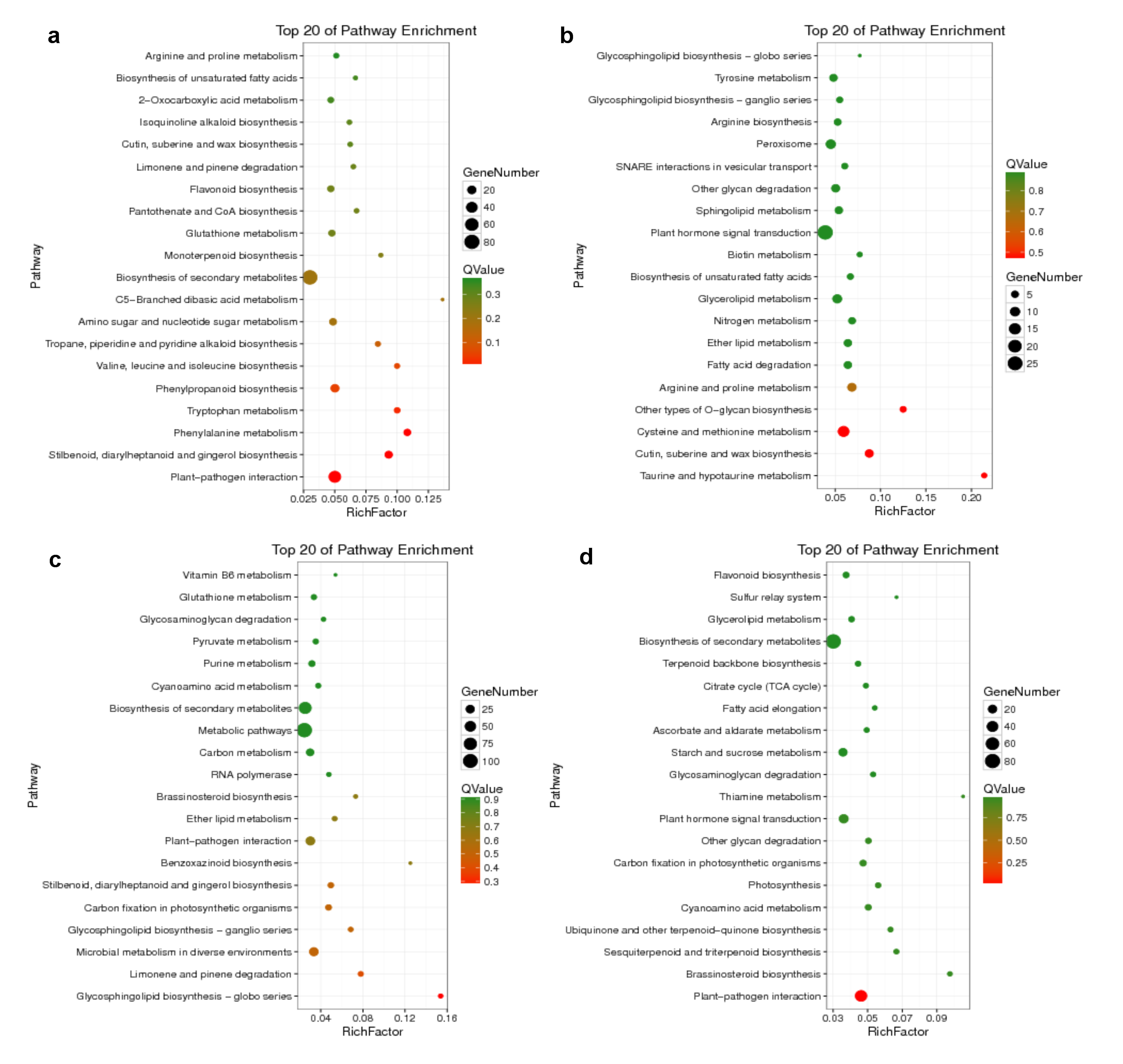

Supplement: Supplementary file 1 — Additional file 1: Figure S1 Growth architectures of MZ (a and d), QQ (b and e) and LYQQ (c and f) in a natural tea garden. The shoots of QQ and LYQQ exhibit a zigzag shape. Figure S2 Stem morphology (a) and inter-node length (b) analysis of MZ, LYQQ and QQ. Mature stems were collected on February 2020. The length of the inter-node between the third and fourth nodes (red lines) was determined (n = 5). ** indicates a significant difference at the 0.01 level. Figure S3 GO enrichment analysis of DEGs identified from the comparisons MZ-vs-QQ (a), MZ-vs-LYQQ (b), and QQ-vs-LYQQ (c). Figure S4 KEGG enrichment analysis of DEGs identified from the comparisons MZ-vs-LYQQ_MZ-vs-QQ _QQ-vs-LYQQ (a), MZ-vs-LYQQ (b), MZ-vs-QQ (c) and QQ-vs-LYQQ (d). Figure S5. Differential metabolites identified from MZ-vs-QQ, MZ-vs-LYQQ, and QQ-vs-LYQQ. [file 12870_2020_2311_MOESM1_ESM.zip › Additional file 1 Fig. S4.tif]

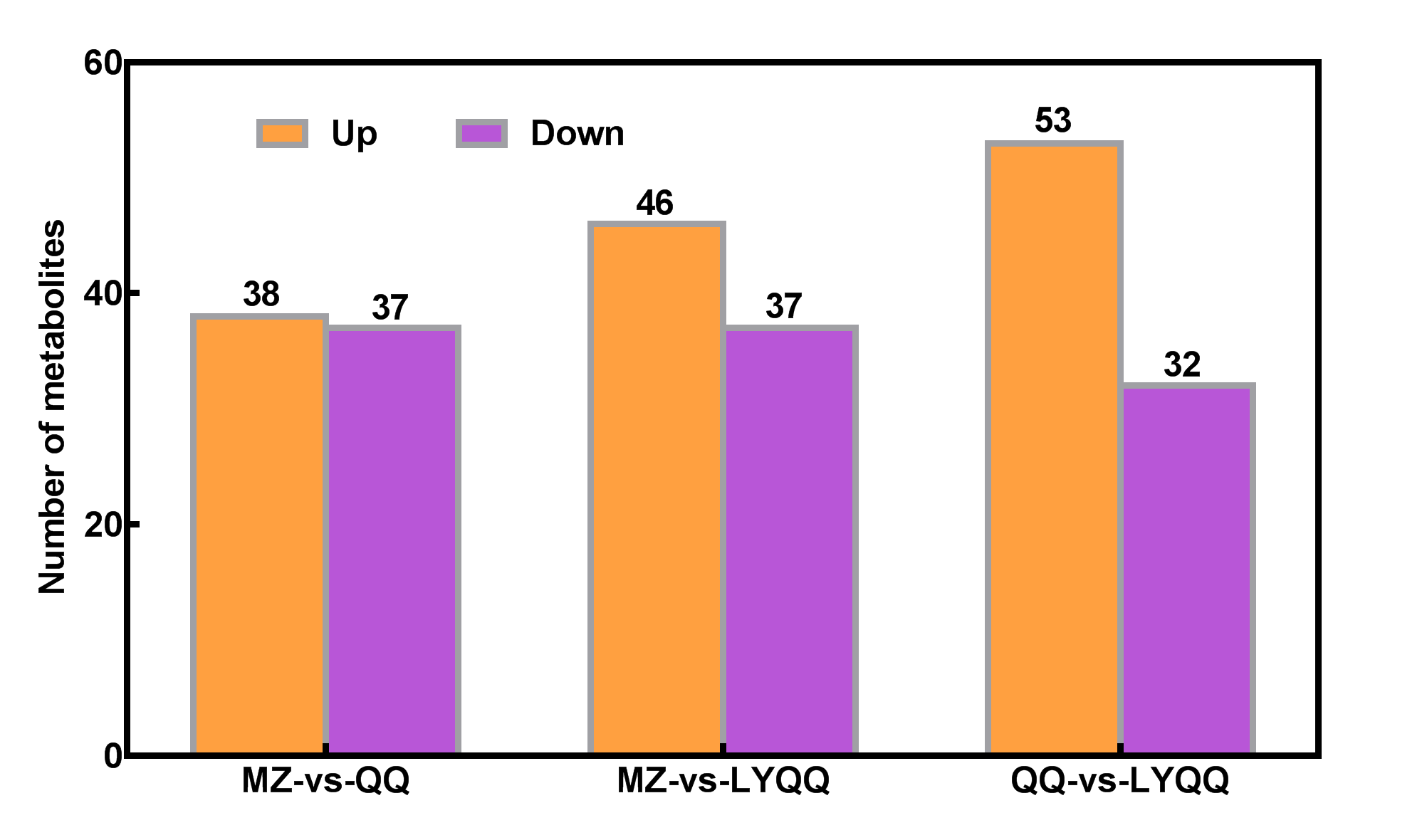

Supplement: Supplementary file 1 — Additional file 1: Figure S1 Growth architectures of MZ (a and d), QQ (b and e) and LYQQ (c and f) in a natural tea garden. The shoots of QQ and LYQQ exhibit a zigzag shape. Figure S2 Stem morphology (a) and inter-node length (b) analysis of MZ, LYQQ and QQ. Mature stems were collected on February 2020. The length of the inter-node between the third and fourth nodes (red lines) was determined (n = 5). ** indicates a significant difference at the 0.01 level. Figure S3 GO enrichment analysis of DEGs identified from the comparisons MZ-vs-QQ (a), MZ-vs-LYQQ (b), and QQ-vs-LYQQ (c). Figure S4 KEGG enrichment analysis of DEGs identified from the comparisons MZ-vs-LYQQ_MZ-vs-QQ _QQ-vs-LYQQ (a), MZ-vs-LYQQ (b), MZ-vs-QQ (c) and QQ-vs-LYQQ (d). Figure S5. Differential metabolites identified from MZ-vs-QQ, MZ-vs-LYQQ, and QQ-vs-LYQQ. [file 12870_2020_2311_MOESM1_ESM.zip › Additional file 1 Fig. S5.tif]
