# Supplementary material for: Transcriptome and metabolite analyses provide insights into zigzag-shaped stem formation in tea plants (Camellia sinensis)
Source: BMC Plant Biol. 2020 Mar 4;20:98. doi: 10.1186/s12870-020-2311-z (PMC7057490; doi:10.1186/s12870-020-2311-z)
Supplement: Supplementary file 2 — Additional file 2: Table S1 Summary of the RNA-Seq data derived from MZ, QQ, and LYQQ. Table S2 Statistics of the number of detected genes in each cultivar. [file 12870_2020_2311_MOESM2_ESM.zip › Additional file 2 Table S1.docx]

**Table S1** Summary of the RNA-Seq data derived from MZ, QQ, and LYQQ

| Sample | Clean Reads Number (million) | HQ Clean Reads Number (million) | Clean Reads Q20 (%) | Clean Reads Q30 (%) | Clean Reads GC (%) | Clean Reads Ratio (%) | Mapping Ratio (%) |
| --- | --- | --- | --- | --- | --- | --- | --- |
| QQ1 | 3.91 | 3.82 | 97.49 | 92.48 | 44.55 | 97.69 | 76.22 |
| QQ2 | 4.93 | 4.83 | 97.7 | 92.97 | 44.49 | 97.86 | 76.43 |
| QQ3 | 4.74 | 4.63 | 97.47 | 92.45 | 44.43 | 97.56 | 75.75 |
| LYQQ1 | 5.10 | 4.99 | 97.65 | 92.82 | 44.42 | 97.89 | 75.95 |
| LYQQ2 | 4.65 | 4.54 | 97.39 | 92.26 | 44.4 | 97.68 | 75.51 |
| LYQQ3 | 6.07 | 5.95 | 97.66 | 92.85 | 44.38 | 98.01 | 75.98 |
| MZ1 | 5.01 | 4.92 | 97.81 | 93.18 | 44.47 | 98.09 | 76.32 |
| MZ2 | 5.55 | 5.45 | 98.29 | 94.47 | 44.52 | 98.2 | 77.13 |
| MZ3 | 6.10 | 6.00 | 98.55 | 95.07 | 44.53 | 98.42 | 77.93 |
| Total | 46.06 | 45.13 |  |  |  |  |  |
