# Supplementary material for: Transcriptome and metabolite analyses provide insights into zigzag-shaped stem formation in tea plants (Camellia sinensis)
Source: BMC Plant Biol. 2020 Mar 4;20:98. doi: 10.1186/s12870-020-2311-z (PMC7057490; doi:10.1186/s12870-020-2311-z)
Supplement: Supplementary file 2 — Additional file 2: Table S1 Summary of the RNA-Seq data derived from MZ, QQ, and LYQQ. Table S2 Statistics of the number of detected genes in each cultivar. [file 12870_2020_2311_MOESM2_ESM.zip › Additional file 2 Table S2.docx]

**Table S2** Statistics of the number of detected genes in each cultivar

| Cultivar | All Gene Number | New Gene Number | Known Gene Number | Known Gene Number (%) |
| --- | --- | --- | --- | --- |
| MZ | 34374 | 6353 | 28021 | 82.58 |
| LYQQ | 34248 | 6266 | 27982 | 82.46 |
| QQ | 33598 | 6157 | 27441 | 80.87 |
