# Supplementary material for: Transcriptome and metabolite analyses provide insights into zigzag-shaped stem formation in tea plants (Camellia sinensis)
Source: BMC Plant Biol. 2020 Mar 4;20:98. doi: 10.1186/s12870-020-2311-z (PMC7057490; doi:10.1186/s12870-020-2311-z)
Supplement: Supplementary file 3 — Additional file 3: Table S3 Seventy-six key DEGs identified to be involved in zigzag-shaped stem formation in tea plants. [file 12870_2020_2311_MOESM3_ESM.docx]

**Table S3** Seventy-six key DEGs identified to be involved in zigzag-shaped stem formation in tea plants

| Genes id | Log2 value of fold change | | Symbol | Description | Function |
| --- | --- | --- | --- | --- | --- |
|  | MZ-vs-QQ | MZ-vs-LYQQ |  |  |  |
| TEA005738.1 | -1.34 | -1.60 | At2g32990 | Endoglucanase 11 | Cell wall synthesis and cell expansion |
| TEA030545.1 | 1.73 | 1.49 | CSLG1 | Cellulose synthase-like protein |  |
| TEA032164.1 | 1.86 | 1.00 | CSLG3 | Cellulose synthase-like protein |  |
| TEA022978.1 | 4.54 | 5.33 | CTL1 | Chitinase-like protein |  |
| TEA012391.1 | -2.25 | -2.54 | EXLB1 | Expansin-like |  |
| TEA027164.1 | 2.05 | 1.86 | EXPA8 | Expansin-A8 like |  |
| TEA030087.1 | -1.48 | -1.51 | INVA | Invertase 5 |  |
| XLOC_006586 | 3.11 | 4.01 | LRX1 | Leucine-rich repeat extensin-like protein 1 |  |
| TEA006408.1 | -1.97 | -1.37 | Nefh | Proline-rich cell wall protein |  |
| TEA026842.1 | -9.08 | -7.49 | PECS-2.1 | Pectinesterase |  |
| TEA004581.1 | 9.69 | 9.74 | PME2.1 | Pectinesterase |  |
| XLOC_003301 | 1.51 | 1.87 | PME8 | Pectinesterase |  |
| XLOC_021264 | -10.82 | -3.47 | RWA2 | REDUCED WALL ACETYLATION 2 |  |
| TEA020219.1 | -3.96 | -3.37 | UGT74B1 | UDP-glycosyltransferase 74B5 |  |
| TEA019643.1 | -3.47 | -2.63 | XTH16 | Xyloglucan endotransglucosylase/hydrolase |  |
| TEA031643.1 | -1.95 | -1.82 | XTH22 | Xyloglucan endotransglucosylase/hydrolase protein 22 |  |
| XLOC_007313 | -11.27 | -11.27 | XTH8 | Xyloglucan endotransglucosylase/hydrolase protein 8 |  |
| TEA006962.1 | -1.13 | -1.76 | Xyl2 | Beta-xylosidase/alpha-L-arabinofuranosidase 2 |  |
| XLOC_040461 | -11.48 | -5.10 | ISA3 | Isoamylase 3 |  |
| XLOC_038810 | -2.43 | -1.87 | ARF4 | Auxin response factor 2 | Phytorhormone |
| XLOC_034141 | -1.09 | -1.78 | BAMT | Salicylic acid carboxyl methltransferase |  |
| TEA026305.1 | -2.13 | -1.26 | GH3.11 | Auxin early response protein GH3.9 |  |
| TEA020186.1 | 2.85 | 1.95 | GH3.5 | Jasmonic acid-amido synthetase |  |
| TEA025180.1 | -2.93 | -2.56 | GH3.6 | Indole-3-acetic acid-amido synthetase |  |
| XLOC_043245 | -2.43 | -12.05 | IAA13 | Auxin-responsive protein IAA13 |  |
| TEA031962.1 | -1.73 | -1.81 | IAMT1 | Indole-3-acetate O-methyltransferase 1 |  |
| TEA005980.1 | 1.54 | 1.75 | ARF17 | Auxin response factor like |  |
| TEA012728.1 | -1.75 | -2.54 | PIN | PIN-LIKES |  |
| TEA019069.1 | -8.11 | -8.11 | PIN3 | protein PIN-LIKES 3-like |  |
| TEA001395.1 | -9.86 | -9.86 | SEC11A | Signal peptidase complex catalytic subunit SEC11A | Vesicular trafficking |
| XLOC_004426 | 1.37 | 2.36 | SEC13B | Signal peptidase complex catalytic subunit SEC13B |  |
| XLOC_037235 | 6.26 | 6.40 | SEC22 | 25.3 kDa vesicle transport protein |  |
| TEA030236.1 | 1.67 | 1.41 | SEC6 | Exocyst complex component SEC6 |  |
| XLOC_057225 | 1.48 | 2.24 | SECA2 | Signal peptidase complex catalytic subunit SECA2 |  |
| TEA008155.1 | -5.80 | -6.53 | SEC1 | SEC1 family transport protein |  |
| XLOC_031693 | 4.14 | 4.28 | VAMP714 | Vesicle-associated membrane protein 714 |  |
| TEA007337.1 | -9.70 | -9.70 | vps18 | Vacuolar protein sorting-associated protein 18 |  |
| TEA031089.1 | -4.88 | -10.20 | VPS41 | Vacuolar protein sorting-associated protein 41 |  |
| XLOC_036914 | 5.97 | 6.26 | KMS1 | Vacuole membrane protein KMS1 |  |
| TEA021222.1 | 1.52 | 1.46 | VSR6 | Vacuolar-sorting receptor 6 |  |
| TEA017728.1 | -10.94 | -10.94 | AGL61 | Floral homeotic protein APETALA 1 | Transcription factors |
| TEA012041.1 | -12.47 | -12.47 | TIFY | Protein TIFY |  |
| TEA026168.1 | -11.61 | -11.61 | ONAC010 | NAC transcription factor ONAC010 |  |
| XLOC_053049 | -11.18 | -11.18 | AP2 | Transcription factor APETALA2 |  |
| TEA032867.1 | -11.08 | -11.08 | WOX2 | WUSCHEL-related homeobox 2 |  |
| TEA027175.1 | -10.98 | -10.98 | TINY | Ethylene-responsive transcription factor TINY |  |
| TEA026206.1 | -10.89 | -10.89 | MYB1R1 | Transcription factor MYB1R1 |  |
| TEA003577.1 | -12.75 | -6.84 | SPL14 | Squamosa promoter-binding-like protein |  |
| TEA030941.1 | -2.02 | -3.52 | HEC1 | Transcription factor HEC1 |  |
| TEA000681.1 | -2.76 | -3.16 | BHLH18 | Transcription factor bHLH18 |  |
| TEA006216.1 | -2.15 | -1.70 | SPT | Transcription factor SPATULA |  |
| TEA022970.1 | -2.05 | -1.39 | GRF1 | Growth-regulating factor 1 |  |
| TEA031877.1 | 2.75 | 2.27 | MYC4 | Transcription factor bHLH041 |  |
| TEA009726.1 | 1.69 | 2.35 | IBH1 | Transcription factor IBH1-like |  |
| TEA023233.1 | 1.90 | 2.55 | WRKY28 | WRKY transcription factor 28 |  |
| TEA031729.1 | 2.28 | 2.80 | DIVARICATA | Transcription factor DIVARICATA |  |
| TEA022287.1 | 3.84 | 3.93 | JUB1 | Transcription factor JUNGBRUNNEN 1 |  |
| TEA030046.1 | -12.77 | -12.77 | SCL3 | Scarecrow-like protein |  |
| TEA009512.1 | -10.04 | -10.04 | ASK9 | Shaggy-related protein kinase | Others |
| TEA031847.1 | -4.61 | -10.81 | LAZY | LAZY protein |  |
| TEA001744.1 | -5.22 | -5.47 | LAZY1 | LAZY1-like protein |  |
| TEA000822.1 | -10.39 | -3.70 | PMA3 | Plasma membrane ATPase 3 |  |
| XLOC_028596 | -6.53 | -3.88 | RECA | DEFECTIVE IN MERISTEM SILENCING 3 |  |
| XLOC_032980 | -10.69 | -3.63 | RER3 | RETICULATA-RELATED 3 |  |
| TEA031914.1 | 3.49 | 3.12 | WAK5 | Wall-associated receptor kinase |  |
| XLOC_005127 | -3.96 | -10.16 | WAKL9 | Wall-associated kinase family protein |  |
| TEA013725.1 | 5.38 | 6.17 | WAT1-R | WAT1-related protein |  |
| XLOC_017186 | 9.02 | 10.02 | WAT1-R | WAT1-related protein At1g44800-like |  |
| TEA030753.1 | -3.62 | -9.95 | VLN2 | VILLIN2 protein |  |
| TEA013639.1 | -7.57 | -7.57 | ADF2 | actin-depolymerizing factor 2 |  |
| XLOC_028345 | -4.51 | -2.51 | TPL | Topless-like |  |
| TEA008751.1 | -4.53 | -4.21 | TPR1 | Topless-related protein |  |
| TEA025988.1 | -1.28 | -1.65 | CDC20-1 | Cell division cycle 20.1, cofactor of APC complex-like |  |
| TEA031938.1 | -2.74 | -2.15 | CDC6B | Cell division control protein 6 B |  |
