# Supplementary material for: Transcriptome and metabolite analyses provide insights into zigzag-shaped stem formation in tea plants (Camellia sinensis)
Source: BMC Plant Biol. 2020 Mar 4;20:98. doi: 10.1186/s12870-020-2311-z (PMC7057490; doi:10.1186/s12870-020-2311-z)
Supplement: Supplementary file 4 — Additional file 4: Table S4 DEGs and primers used for qRT-PCR validation of the transcriptome. [file 12870_2020_2311_MOESM4_ESM.docx]

**Table S4** DEGs and primers used for qRT-PCR validation of the transcriptome

| DEGs id | Forward primer | Reverse primer |
| --- | --- | --- |
| TEA000001.1 | GCTGGAACAGCAGAAACGTCTC | CCTGAGTCTAGCCAGTTTCCA |
| TEA000010.1 | TGGTCAGTGGTGGTGCAGATC | CCGACGCCTCGCTTGCATCGC |
| TEA000022.1 | GCGATCGCGTGAGGCTGCT | GGGAGATCACAGAGAATCC |
| TEA000030.1 | GGAAGGGAAAGCAATCCAATC | CGAGTATGAGGTCGGTGTC |
| TEA000038.1 | CCTGTGCCACCTGCACCGACG | GCCTGAAACGCCATTCATT |
| TEA000048.1 | CAGAGGACATGGCCTATCCA | GATCTACCCCTATTGCAATCTC |
| TEA000051.1 | CGGCTGCTCTCTTGCAAGGAAT | GTAGATGTTCTGGTTGATTGT |
| TEA000192.1 | GATGCGGTTCAGAAGTCAGG | ACGCCAGTTCCATCCATGT |
| TEA000234.1 | GACACAGTGGGAGTTGGATG | CCTGTTCTACTGCCATCC |
| XLOC_037486 | TCACATGGAGTTACGGTGCC | TCACACTCCTGTGGCAAGTC |
| XLOC_038013 | CAACATCTCTTCAGCCTCTTCCA | CAGGAGCAGAAAGAGCCTGCG |
| XLOC_039015 | GGACTCATGAGATGGGCCACC | CTACAACTCTTTCCACACC |
| TEA012676.1 | TAGGTCGGCTAACTCAGATGA | CAGTAAAGTCTCTCTCGAGCTCG |
| TEA012789.1 | CATTTGATCCAGCAAAGAAGCGTG | CCATATTCTTCCATTACC |
| TEA013024.1 | CCTCAGGAAGTGGGTTCAAGTC | GTCTGCAATGTGTATGGCT |
| TEA000388.1 | TTGTGCCTGTTGACATGAAT | TCTAGATATGTGACGTGCA |
